# Efficacy of oral moxidectin against susceptible and resistant isolates of *Dirofilaria immitis* in dogs

**DOI:** 10.1186/s13071-017-2429-5

**Published:** 2017-11-09

**Authors:** Tom L. McTier, Robert H. Six, Aleah Pullins, Sara Chapin, John W. McCall, Douglas Rugg, Steven J. Maeder, Debra J. Woods

**Affiliations:** 1 Zoetis, Veterinary Medicine Research and Development, Kalamazoo, MI USA; 2 TRS Labs, Athens, GA USA

**Keywords:** Moxidectin, Oral, Heartworm, *Dirofilaria immitis*, Macrocyclic lactone, Resistance, Resistant isolate, Prevention

## Abstract

**Background:**

Monthly topical and sustained-release injectable formulations of moxidectin are currently marketed; however, an oral formulation, while approved at a dose of 3 μg/kg, is not currently marketed in the United States. Although resistance of heartworms to all macrocyclic lactone (ML) heartworm preventives (ivermectin, milbemycin, selamectin and moxidectin) has been demonstrated, to date no data have been reported on the effectiveness of oral moxidectin against recent isolates of *Dirofilaria immitis*.

**Methods:**

A total of nine studies were conducted to determine the efficacy of moxidectin against a range of older and recently sourced heartworm isolates. Dogs (groups of three to eight) were inoculated with 50 *D. immitis* infective larvae (L3) from nine different isolates (MP3, Michigan, JYD-34, ZoeMO-2012, ZoeKy-2013, ZoeLA-2013, GCFL-2014, AMAL-2014 and ZoeAL-2015) and treated 28–30 days later with single oral doses of 3 μg/kg of moxidectin. Additionally, one group of dogs that was inoculated with JYD-34 was treated monthly for 3 consecutive months beginning 30 days post inoculation. Dogs were held for approximately 4 months after the initial (or only) treatment and then necropsied for recovery of adult heartworms.

**Results:**

A single dose of 3 μg/kg of moxidectin was 100% effective in preventing the development of five of nine heartworm isolates (MP3, Michigan, ZoeKy, GCFL and ZoeAL isolates), confirming their susceptibility to oral moxidectin at this dose. MP3 and Michigan are isolates sourced from the field more than 9 years ago, while ZoeKy, ZoeAL and GCFL were isolated from the field within the past 2 to 3 years. Against JYD-34, ZoeMO, ZoeLA and AMAL isolates, a single dose of 3 μg/kg of moxidectin was not completely effective, with efficacies of 19%, 82%, 54% and 62%, respectively, demonstrating resistance of these heartworm isolates to oral moxidectin at this dosage. Three consecutive monthly doses of 3 μg/kg of moxidectin were also incompletely effective against the JYD-34 isolate, with an efficacy of 44%. JYD-34 was originally isolated in 2010, while ZoeMO, ZoeLA and AMAL were isolated within the past 2 to 3 years.

**Conclusions:**

A single oral dose (3 μg/mg) of moxidectin was 100% effective in preventing the development of ML-susceptible heartworm isolates while being incompletely effective against ML-resistant isolates.

## Background

Moxidectin, a macrocyclic lactone (ML), is used in a number of products available for prevention of heartworm (*Dirofilaria immitis*) disease in dogs and cats in the United States and other markets. The products currently marketed in the United States are a monthly topical and a 6-month, sustained-release injectable formulation. The initial data supporting moxidectin as a heartworm preventive were based on an oral formulation that demonstrated very potent activity, with a dose as low as 0.5 μg/kg being 100% effective at preventing the development of *D. immitis* when administered 2 months after inoculation of 50 infective larvae (L3) [1]. In the same paper, a single oral 3 μg/kg dose of moxidectin was reported to be 64% effective when given 3 months post inoculation with L3. Moxidectin was also shown to be 100% effective in preventing the development of heartworms when administered orally at 1 or 3 μg/kg monthly or 3 μg/kg bimonthly when dogs were exposed to natural heartworm infection in Georgia and Louisiana [2]. The 3 μg/kg oral dose was approved for use as a preventive but was never marketed in the United States. It is, however, approved and sold in some Asia Pacific markets. All of the original work with oral moxidectin was conducted with a single heartworm isolate (UGA) that had been maintained under laboratory conditions at TRS Labs (Athens, Georgia, USA) for a number of years prior to use in the moxidectin program (John McCall, personal oral communication, October 2016). This same isolate had also been used previously to assess the efficacy of oral ivermectin for heartworm prevention. Resistance of heartworm to MLs is becoming an increasing concern, with reports of resistance of at least one isolate to all currently marketed active MLs [3–5]. To date, however, no data have been reported on the effectiveness of oral moxidectin against recent isolates of *D. immitis.* The objective of the current study was to assess the efficacy of 3 μg/kg moxidectin, administered orally, in preventing the development of *D. immitis* isolates collected from various sources over the previous10 years.

## Methods

### Ethical approval

The studies were masked, negative placebo-controlled, randomized laboratory efficacy studies conducted in Georgia and Michigan, USA. Study procedures were conducted in accordance with the VICH (GL19) guidelines [6]. Masking of the studies was assured through the separation of functions. All personnel conducting observations or animal care or performing infestations and counts were masked to treatment allocation. All protocols for these studies were approved by the appropriate animal welfare committees or governing authorities, and studies were conducted in accordance with state and national/international regulations regarding animal welfare.

### Heartworm isolates

The nine heartworm isolates used in these studies had been maintained in the laboratory for several years (Michigan, MP3 or JYD-34) or recently acquired (within the previous 4 years) by Zoetis (ZoeMO-2012, ZoeKY-2013, ZoeAL-2015, ZoeLA-2013, GCFL-2014 and AMAL-2014). These isolates were acquired from various sources (individual client-owned animals, humane societies, or private kennels) primarily located in the southeastern United States (Table 1). Microfilaremic blood from individual heartworm-infected animals was collected and sent overnight to either TRS Labs or Zoetis. *Aedes aegypti* mosquitoes (black-eyed Liverpool strain) were fed the blood, and mosquitoes were held for ~15 days to allow the microfilariae (MF) to develop to the infective stage. Infective larvae (L3) were then collected from the mosquitoes and inoculated into recipient dogs (40–50 L3 per dog) to establish a new infection [7]. Dogs were held to allow maturation of the heartworms and for the adult worms to begin producing MF. The MF from these recipient animals were then used to infect mosquitoes from which L3 were collected to inoculate study animals with the isolates using the methods described earlier [7].

**Table 1:** Heartworm isolate details for nine different heartworm (*D. immitis*) isolates used to assess the preventive efficacy of 3 μg/kg of oral moxidectin in dogs

*D. immitis* isolate	Year isolated	Isolate location	Responsible for original isolate collection/currently maintained	Comments
MP3	2006	GA (Northeast)	TRS Labs/Zoetis	Refractory, not resistant, published results for other products at use dose <100% [7–10]; original isolate from TRS Labs
Michigan	2007	MI	TRS Labs/Zoetis	Confirmed susceptible isolate >6 years previous from TRS Labs
JYD-34	2010	Pittsville, IL (CHK- Kennel)	TRS Labs/Zoetis	Confirmed ML-resistant isolate from published reports [3, 4]; original isolate from TRS Labs
ZoeMO	2012	Pittsville, IL (CHK- Kennel)	Zoetis/Zoetis	Related to JYD-34, isolated from the same dog as JYD-34 but 2.5 years later. Dog had been kept in mosquito-proof quarters and received no ML preventive or adulticide therapy
ZoeKY	2013	Slayersville, KY (CHK-Kennel)	Zoetis/Zoetis	New isolate; no documented previous medical history
ZoeLA	2013	Zachary, AL (Dr. Lynn Buzhardt)	Zoetis/Zoetis	New isolate from 6-year-old English bulldog, originally from Ellis, AR, moved to Slaughter, LA, at 3 months of age; on Heartgard® for 2 years but no HW prevention for previous 3 years before isolate collection
GCFL	2014	Fort Myers, FL (Gulf Coast Human Society)	Zoetis/Zoetis	New isolate from 3-year-old heartworm-positive pit-bull mix from local humane society; no documented previous medical history
AMAL	2014	Westover, AL (Dr. Jay Crisman)	Zoetis/Zoetis	New isolate from a 3-year-old, heartworm-positive Husky with no previous documented medical history
ZoeAL	2015	Wetumpka, AL (Dr. Jay Crisman)	Zoetis/Zoetis	New isolate from a heartworm-positive 4-year-old pug with no previous ML preventive use

### Animals

Three, six, or eight beagles (males and females), ranging in age from 6 months to 6 years and in body weight from 6.0 to 14.0 kg at the start of the study (Day 0 treatment) were used for these studies (Table 2).

**Table 2:** Study design for nine studies using nine different isolates of *D. immitis* used to assess the efficacy of 3 μg/kg of oral moxidectin in dogs

Study	No. of dogs/Gp^a^	*D. immitis* isolate	Treatment^b^	Dosage (μg/kg)	Day of inoculation	Days of treatment (oral)	Day of necropsy (PI)
1	8	Michigan	Moxidectin	3	−30	0	116 (146)
2	8	MP3	Moxidectin	3	−30	0	120 (150)
3	6	ZoeKY	Moxidectin	3	−30	0	117 (147)
4	3	GCFL	Moxidectin	3	−28	0	120 (148)
5	6	ZoeAL	Moxidectin	3	−28	0	120 (148)
6	8	JYD-34	Moxidectin	3	−30	0	122 (152)
6	8	JYD-34	Moxidectin	3	−30	0, 30 and 60	122 (152)
7	6	ZoeMO	Moxidectin	3	−30	0	117 (147)
8	6	ZoeLA	Moxidectin	3	−30	0	118 (148)
9	3	AMAL	Moxidectin	3	−28	0	120 (148)

^a^Matched placebo control group was included in each study

^b^ProHeart® tablets shaved to deliver the exact dose

### Design

Nine studies were conducted to determine the efficacy of oral moxidectin against nine different isolates of *D. immitis.* Within each study, dogs were randomly allocated to treatments with either placebo or oral moxidectin (3 μg/kg) based on pretreatment body weight. Dogs were inoculated with infective larvae (L3) of the various isolates (MP3, Michigan, ZoeKy, ZoeAL, GCFL, JYD-34, ZoeMO, ZoeLA and AMAL) ~1 month prior to treatment (Table 2).

Twenty-eight to 30 days prior to treatment, each dog was administered 50 viable *D. immitis* L3, of the specific isolate required, by subcutaneous injection in the inguinal region.

### Treatment

Dogs were treated 28 to 30 days after inoculation with single doses of 3 μg/kg of moxidectin, using ProHeart® tablets (commercial products sourced from Australia) shaved to deliver the exact dose. Additionally, in one of the studies, one group of dogs that was inoculated with JYD-34 was treated monthly for 3 consecutive months, beginning 30 days post inoculation.

Feed was withheld overnight prior to dosing. Each dog was offered its regular food ration within approximately 20 min of dosing. Dogs in the control groups were administered a placebo tablet. Dogs in the moxidectin treatment groups were administered ProHeart® moxidectin tablets, which had been shaved, based on each dog’s individual body weight, to deliver exactly 3 μg/kg moxidectin (Table 2). The most recent body weight (within 4 days of treatment) was used to calculate dosage. The tablets were administered by mouth. Swallowing was encouraged (eg, by holding the mouth closed and stroking the neck), and approximately 5 mL of water was administered by mouth via syringe. Each dog was observed for several minutes after dosing for evidence that the dose was swallowed, and for potential adverse events associated with the administration of the tablet (eg, choking, drooling, gagging or vomiting). Dogs were observed approximately 2 h after dosing for any evidence of emesis.

#### Microfilariae and adult heartworm counts

Blood samples from each animal were collected in potassium EDTA tubes prior to inoculation with L3 and ~2 months post treatment. These samples were examined for adult *D. immitis* antigen and for MF. The 2-month posttreatment blood tests were designed to detect existing heartworm infections in dogs when they were acquired prior to selection for the study but were not detectable at that time. All test results were negative for heartworms, indicating no previous infection with heartworms.

Approximately 5 months (146–152 days) post inoculation, all dogs were humanely euthanized. At the time of euthanasia, each dog was given 2 mL of heparin (1000 USP units/mL) intravenously prior to a lethal dose of an approved euthanasia agent. After euthanasia, the pleural and peritoneal cavities were examined for adult *D. immitis* worms, and the posterior and anterior venae cavae were clamped before removal of the heart and lungs. The precava, right atrium, right ventricle, and pulmonary arteries (including those coursing through the lungs) were dissected and examined for worms. The number and viability of worms recovered from each dog was recorded. Only worms that were normal in both appearance and motility were considered live. All other worms were considered dead.

#### Animal observations

On the days of treatment, dogs were assessed for overall health prior to treatment and at.

1 (±15 min), 3 (±30 min), and 6 (±1) hours after treatment administration and again at 24 (±1) hours after treatment administration. Additionally, for the remainder of the study dogs were observed twice daily for general health.

## Results

As mentioned previously, some of the isolates tested in these studies have been maintained in the laboratory for several years and have been used to test the efficacy of other MLs and heartworm preventive products (Michigan, MP3 and JYD-34). The other isolates (ZoeKY, ZoeAL, GCFL, ZoeMO, ZoeLA and AMAL) were recently isolated, and no data were available on the efficacy of any ML against them.

Moxidectin, administered at 3 μg/kg orally as ProHeart® tablets, was 100% effective against the following isolates: Michigan, MP3, ZoeKY, GCFL and ZoeAL (Table 3). All placebo-treated control dogs in all studies had adult heartworms at necropsy, with a range of 16 to 47 worms recovered from these dogs and a mean across all five studies of 29.4 worms/dog (average recovery of L3 inoculated, 58.8%). These represent adequate challenge recoveries for all studies and differences between mean recoveries in the placebo and moxidectin-treated groups was statistically significant for all five studies (*P* < 0.05). These data indicate susceptibility of these isolates to moxidectin at this dose and confirm the general susceptibility of heartworm to moxidectin at this dose rate.

**Table 3:** Efficacy of oral moxidectin (3 μg/kg) in preventing the development of selected isolates of *D. immitis*

						Adult *D. immitis* worm counts^1^			
Study	*D. immitis* isolate	Treatment^2^	Dosage (μg/kg)	Days of Tx	No. Dogs with worms	Worm count range	Geometric mean^3^	% reduction	Isolate phenotype
1	Michigan	Placebo	NA	0	8 of 8	18–32	24.4^a^	NA	
1	Michigan	Moxidectin	3	0	0 of 8	0	0.0^b^	100	Susceptible
2	MP3	Placebo	NA	0	8 of 8	21–42	35.1^a^	NA	
2	MP3	Moxidectin	3	0	0 of 8	0	0.0^b^	100	Susceptible
3	ZoeKY	Placebo	NA	0	6 of 6	16–36	26.2^a^	NA	
3	ZoeKY	Moxidectin	3	0	0 of 6	0	0 ^b^	100	Susceptible
4	GCFL	Placebo	NA	0	3 of 3	21–35	28.4^a^	NA	
4	GCF	Moxidectin	3	0	0 of 3	0	0^b^	100	Susceptible
5	ZoeAL	Placebo	NA	0	6 of 6	26–47	33.0^a^	NA	
5	ZoeAL	Moxidectin	3	0	0 of 6	0	0^b^	100	Susceptible
6	JYD-34	Placebo	NA	0, 30, 60	8 of 8	29–43	35.9^a^	NA	
6	JYD-34	Moxidectin	3	0	8 of 8	20–39	29.1^b^	19.0	Resistant
6	JYD-34	Moxidectin	3	0, 30, 60	8 of 8	7–36	20.0^b^	44.4	Resistant
7	ZoeMO	Placebo	NA	0	6 of 6	15–32	21.2^a^	NA	
7	ZoeMO	Moxidectin	3	0	6 of 6	2–7	5.5^b^	82.7	Resistant
8	ZoeLA	Placebo	NA	0	6 of 6	22–38	32.2^a^	NA	
8	ZoeLA	Moxidectin	3	0	6 of 6	11–26	14.8^b^	54.0	Resistant
9	AMAL	Placebo	NA	0	3 of 3	43–48	45.0^a^	NA	
9	AMAL	Moxidectin	3	0	3 of 3	12–25	17.3^b^	61.6	Resistant

^1^Dogs inoculated with 50 infective larvae (L3) on Days −28 to −30

^2^Moxidectin administered as ProHeart® tablets shaved to deliver the exact dose

^3^Within a study, means with different superscripts are significantly different (*P* < 0.05)

Efficacy against JYD-34 was 19% when administered as a single 3 μg/kg dose 30 days following inoculation and 44% after three consecutive monthly doses at 30, 60, and 90 days following larval inoculation (Table 3). A single 3 μg/kg oral dose of moxidectin was 82.7%, 54.0%, and 61.6% effective against three recently sourced isolates, ZoeMO, ZoeLA, and AMAL, respectively. All moxidectin-treated dogs had heartworms at necropsy with a range of 2 to 7 for ZoeMO and 11–26 for ZoeLA and AMAL. As discussed previously for susceptible isolates, all placebo-treated dogs had heartworms at necropsy, with a range of 11 to 48 worms (mean 33.6) across the four studies. There were exceptionally high worm recoveries in the AMAL placebo animals (Study 9) with 90% (mean 45.0) of the inoculated larvae recovered. This gave assurance that moxidectin-treated dogs received an adequate challenge, and results of these studies can be viewed with confidence. All moxidectin treatment group means were significantly different statistically from their matched placebo group means for all four studies (*P* < 0.05). There was no statistical difference between the two JYD-34 treatment group means in Study 6. These data strongly suggest that these isolates (JYD-34, ZoeLA and AMAL) are ML resistant.

## Discussion

The Michigan isolate is a previously tested isolate known to be susceptible to other MLs at their respective approved use doses [8] (John McCall, personal oral communication, October 2016). Previously published data for the MP3 isolate indicated <100% efficacy of some approved single doses of ML preventives (milbemycin oxime, selamectin, and ivermectin) against this isolate [9, 10]. A single topical dose of moxidectin (2.5 mg/kg) in a formulation also containing imidacloprid (10 mg/kg) was 100% effective [9], and three successive doses of milbemycin oxime (0.5 mg/kg) in a formulation also containing spinosad was also 100% effective against this isolate [8]. The complete effectiveness of a single oral dose of 3 μg/kg of moxidectin against the MP3 isolate, as reported in this paper, underscores the high potency of moxidectin against susceptible/refractory isolates of *D. immitis.* In addition these data also support the conclusions of others [8, 10] who have suggested that MP3 is not a resistant strain of heartworm but may be less susceptible (refractory) than the original isolate (UGA) used to test and gain the original approval of most of the original ML preventive medications. This is further supported by work done by Prichard et al. [11] and reported in this volume, with genetic analysis indicating that MP3 has a genetic profile similar to those isolates that are susceptible to MLs.

A previous report demonstrated that three consecutive monthly doses of either selamectin (6 mg/kg), milbemycin oxime (0.5 mg/kg), or ivermectin (6 μg/kg) were 29%, 52%, and 29% effective, respectively, in preventing the development of the JYD-34 isolate when dogs were inoculated 30 days prior to initial treatment with 50 *D. immitis* L3_,_ with eight of eight dogs in each of the groups having worms (mean range 8.8–13.1). In contrast, a single dose of moxidectin at 2.8 to 6.7 mg/kg applied topically once, 30 days post infection, was 100% effective [3]. The efficacy (44%) of three consecutive monthly doses of 3 μg/kg of oral moxidectin against the JYD-34 isolate in the current study was comparable to that observed previously for the other oral MLs when also given for 3 consecutive months [3].

The difference in efficacy response of JYD-34 (19%) and ZoeMO (83%) to a single 3 μg/kg dose of moxidectin is interesting, and suggests that both of these isolates are ML resistant. As indicated in Table 1, JYD-34 and ZoeMO are related isolates. ZoeMO was originally isolated from the same dog as JYD-34 but 2.5 years after the original JYD-34 isolate was taken. The obvious question is: what changed in the 2.5 years between when JYD-34 and ZoeMO were isolated to cause such a difference in efficacy response? The source dog was held in mosquito-proof quarters and received no additional heartworm inoculations or preventive or adulticidal treatments during the 2.5 years.

There are several possible explanations: (1) if resistant worms are less fit, some of these worms may have died during the intervening period, leaving more susceptible worms to produce more susceptible MF; or (2) without drug pressure, the more susceptible worms may have produced more susceptible MF; or (3) a combination of both. These questions of fitness of resistant worms and the potential impact on resistance phenotypes in populations and implications for resistance spread need further consideration and study.

Prichard et al. [11] have genetically characterized all nine of the isolates that we characterized phenotypically in these studies and their data support our categorizations of susceptible and resistant isolates based on efficacy testing. Additional work with well- characterized isolates will be needed to confirm the best combination of single nucleotide polymorphism (SNP) changes that may be able to better predict ML resistance.

**Fig. 1:**
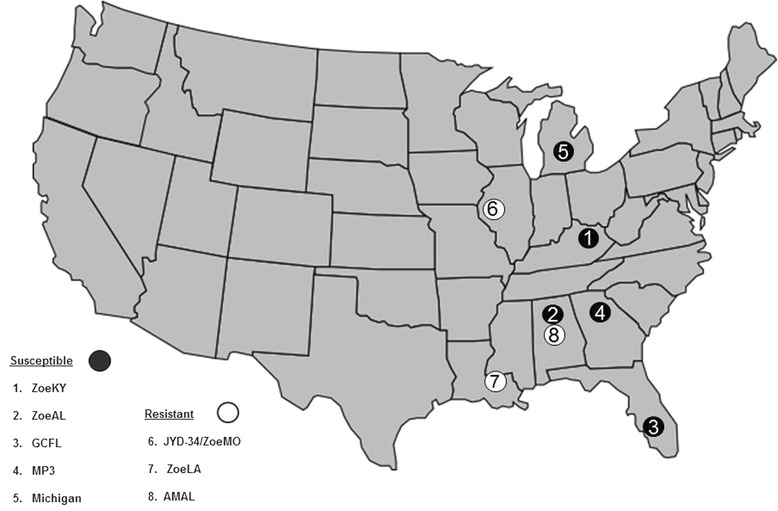
Original locations of the microfilariae-positive dogs used to source heartworm macrocyclic lactone susceptible and resistant isolates collected from 2006 to 2015

Finally, at Zoetis we selected the ZoeLA, ZoeAL, AMAL, and GCFL isolates at random over a period of 3 years (2013–2015), and two of these were determined to be resistant. While this is not a scientifically robust survey due to limited numbers and limited geographical distribution, the finding of two resistant isolates (ZoeLA and AMAL) from randomly selected veterinary patients not under ML selection pressure is a cause for concern. One of these isolates was from the middle of the Mississippi Delta region (Slaughter, Louisiana); however, the other isolate was from just outside the Delta (Westover, Alabama) (Fig. 1). Efforts are ongoing to gather baseline survey data on the prevalence and range of heartworm resistance based on genetic marker correlation with microfilaricidal reduction after ML treatment [12]. Certainly, from the limited number of isolates for which we have data available, there does appear to be a concentration of ML-resistant isolates in and around the Delta region. We should consider this information as we determine how to move forward with a more thorough investigation of the prevalence of heartworm resistance; gain a better understanding of resistance and the factors contributing to its development, maintenance and spread; and begin to discuss options for better management of resistance.

## Conclusions

Characterization of heartworm isolates using a single oral dose of 3 μg/kg of oral moxidectin revealed older and newly acquired field isolates (MP3, Michigan, ZoeKy, GCFL and ZoeAL) that were ML-susceptible, with 100% efficacy in preventing the development of these isolates at this dose. Other ML-resistant isolates (JYD-34, ZoeMO, ZoeLA and AMAL) were also identified that yielded less than complete preventive efficacy when administered as a single 3 μg/kg dose. These data confirm previous published reports that resistance to ML heartworm preventives is real and that additional investigation is needed to further understand various aspects of this resistance.

## Abbreviations

MF: Microfilariae
ML: Macrocyclic lactone
SNP: Single nucleotide polymorphism
